# Revealing the Effects of Methoxy Polyethylene Glycol Ether on the Performance of Polycarboxylate Superplasticizers and Their Desensitization Functions in Relation to Concrete

**DOI:** 10.3390/ma18040772

**Published:** 2025-02-10

**Authors:** Yongqi Da, Longgang Yu, Tingshu He, Zihan Zheng

**Affiliations:** College of Materials Science and Engineering, Xi’an University of Architecture and Technology, Xi’an 710055, China; ylg@xauat.edu.cn (L.Y.); hetingshu@xauat.edu.cn (T.H.); zhengzihan@xauat.edu.cn (Z.Z.)

**Keywords:** polycarboxylate superplasticizer, fluctuations in concrete mixing water, hydration temperature, ester monomer

## Abstract

During the manufacture of high-strength concrete, its sensitivity to variations inconcrete mixing water, the poor adaptability of cement, and high hydration temperatures are often encountered. Therefore, in this paper, firstly, the esterification reaction of methoxy polyethylene glycol ether (MPEG) and methacrylic acid (MAA) was carried out. According to the different molecular weights of MPEG, three kinds of esterification products (MPEG-MAA) were synthesized. Three kinds of PCE-st were synthesized by free-radical copolymerization of three kinds of MPEG-MAA, ethylene glycol polyethylene glycol ether (EPEG), and acrylic acid (AA), and their effects on the properties of cement paste and concrete were analyzed. The results revealed that when the water–cement ratio increased from 0.25 to 0.31, PCE-st with MPEG with a molecular weight of 600 optimally reduced the water content variation sensitivity and increased the fluidity of cement pastes by only 62 mm, while PCE-st with smaller and larger molecular weights of MPEG increased the fluidity by 94 mm and 80 mm, respectively. As the molecular weight of MPEG increased from 400 to 1200, the hydration temperature decreased from 43.8 °C to 39.5 °C, and the setting time was delayed by about 30 min. Finally, the compressive strength of concrete made with PCE-st was similar to that of the concrete made with commercially available PCE-et0.

## 1. Introduction

Since the 21st century, China’s infrastructure construction has been booming, and concrete is one of the most important building materials in modern infrastructure construction. Concrete has application in construction, roads and bridges [1,2], and water conservancy and other projects [3], but concrete often cannot maintain the ideal water–cement ratio in the construction process. Therefore, a change in the water content of external raw materials will significantly affect the construction workability of concrete. At the same time, the hydration temperature must also be paid more attention to due to the wide application of high-strength concrete in order to ensure the durability of concrete [4,5]. At present, polycarboxylate superplasticizers, as one of the key components affecting the moisture change and hydration temperature of high-strength concrete mixtures [6], are gradually receiving extensive attention from the industry.

The branch chains of polycarboxylate superplasticizers can be categorized based on their functional groups into polycarboxylate ether superplasticizers (PCE-et) and polycarboxylate ester superplasticizers [7,8,9]. PCE-et is economical and widely recognized for its ability to achieve an excellent water reduction rate [10]. However, the high sensitivity of PCE-et that results from the complex interaction between PCE-et and cement particles primarily restricts its further development, hindering its wide applicability and ability to meet strict engineering requirements [11]. Varying moisture contents of sand and gravel [12] may impact the charge state and configuration of PCE-et molecules, eventually affecting the interaction force between them and cement particles. In contrast, polycarboxylate ester superplasticizers have a stable molecular structure [13], different functional groups, and the ability to regulate cement hydration; thus, they have lower sensitivity to variations in mixing water and lower hydration temperatures. However, the low water reduction rate also inhibits their wide applicability. Therefore, overcoming the high sensitivity of PCE-et and improving the water reduction rate of polycarboxylate ester superplasticizers are crucial [14].

To exploit the combined advantages of PCE-et and polycarboxylate ester superplasticizers, an ester–ether copolymerized polycarboxylate superplasticizer (PCE-st) was synthesized via free-radical polymerization by adding appropriate amounts of an ester monomer to an ether monomer [15]. PCE-st has an excellent water reduction rate and low sensitivity [16]. Through chemical synthesis, the molecular structure and properties of PCE-st can be accurately regulated to meet the requirements of concrete engineering for material performance improvement, reducing the sensitivity to water changes [17]. However, existing studies have not compared the effects of the same ester compounds with different relative molecular masses on cement-based materials.

In this study, methoxy polyethylene glycol methacrylates (MPEG1-MAA, MPEG2-MAA, and MPEG3-MAA) were synthesized via the esterification of methacrylic acid (MAA) and methoxy polyethylene glycol ethers (MPEG1, MPEG2, and MPEG3) with relative molecular masses of 400, 600, and 1200, respectively. These products were then polymerized with EPEG and AA to obtain PCE-st1, PCE-st2, and PCE-st3, respectively. Advanced microscopic analyses were performed to understand and reveal their mechanisms in practical applications. Infrared spectroscopy was performed to analyze the molecular structure of PCE-st. Its main chemical constituents and functional groups were determined by comparing the absorption peaks of different bands. The zeta potential revealed that PCE-st exhibits good adsorption performance, based on which its mechanism was further studied. Finally, the effects of ester monomers with different molecular weights on the properties of PCE-st, such as the sensitivity of cement pastes to water content variations, hydration temperature, and concrete workability, were analyzed.

## 2. Raw Materials and Characterization

The raw materials and reagents used for the experiments are shown in Table 1.

**Figure 1:**
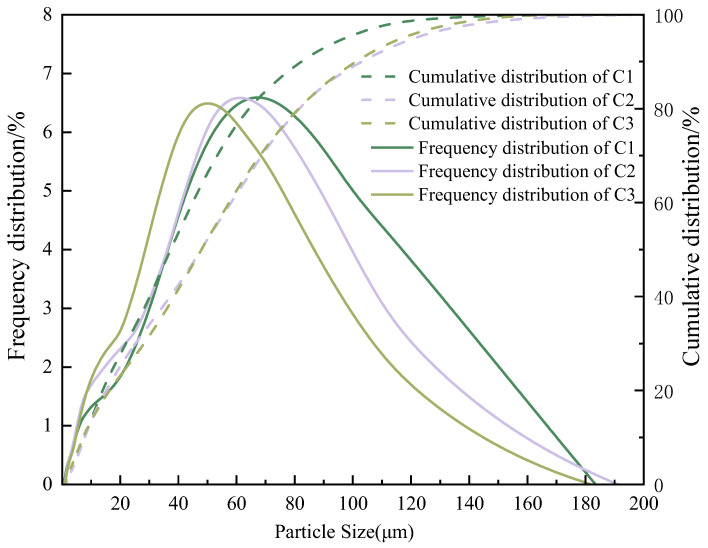
Particle size distribution of all materials.

The following types of cement were used: C1, ordinary Portland cement (P·O 42.5) produced by Hailuo (China, Shaanxi, Qianxian) Group Co, Ltd., with a specific surface area of 356 m^2^/kg; C2, ordinary Portland cement (P·O 42.5) produced by Jidong Heidelberg (China, Shaanxi, Jingyang) Group Co, Ltd., with a specific surface area of 360 m^2^/kg; C3, and ordinary Portland cement (P·O 42.5) produced by Yaobai (China, Shaanxi, Tongchuan) Group Co, Ltd., with a specific surface area of 366 m^2^/kg. The particle size distribution of the three types of cement is shown in Figure 1. Their oxide contents determined via X-ray fluorescence spectrometry are shown in Table 2. Their mortar strengths determined according to GB/T 17671-2021 [18] are shown in Table 3. The aggregate particle size and concrete mix ratio are, respectively, shown in Table 4 and Table 5.

## 3. Synthesis and Characterization of PCE-st

### 3.1. Synthesis of Ester Monomers

Under stirring and heating conditions, MAA was reacted with MPEG1, MPEG 2, and MPEG 3 in a molar ratio of 1.5:1.0. Next, HQ, which accounted for 0.3% of the total mass of the monomer, was added as an inhibitor to prevent unnecessary free-radical polymerization during esterification. PTSA was subsequently added as a catalyst, and its dosage was controlled at 4% of the total mass of the monomer to accelerate esterification. Finally, CH, which accounted for 15% of the total mass of the monomer, was added as a solvent to provide a homogeneous environment for the dissolution of reactants and the reaction.

**Figure 2:**
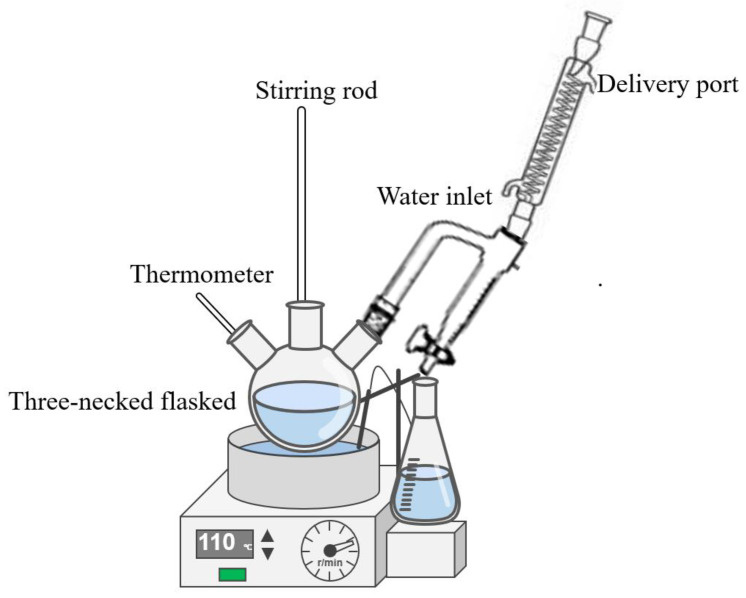
Experimental equipment for ester monomer synthesis.

After all the reactants were completely dissolved, the temperature was increased to 110 °C and maintained for 7 h throughout esterification. This temperature is considered ideal for esterification and achieving a higher conversion rate in a relatively short time. The experimental equipment is shown in Figure 2. The reactants were stirred continuously to ensure an efficient reaction; next, the products were cooled to room temperature, and esterified macromonomers were obtained: MPEG1-MAA, MPEG2-MAA, and MPEG3-MAA.

### 3.2. Synthesis of PCE-st

A certain amount of EPEG was accurately weighed and slowly dissolved in a three-necked flask. Next, an appropriate amount of H_2_O_2_ was added to the solution. Two different solutions were simultaneously prepared: solution A contained a mixture of MPEG1-MAA, MPEG2-MAA, and MPEG3-MAA, with AA in a certain proportion, while solution B contained VC and MPA. VC, a widely used free-radical polymerization initiator, acted as a reducing agent. VC and hydrogen peroxide undergo a redox reaction and generate free radicals that initiate polymerization. MPA, with its unique chain transfer ability, plays a regulatory role during polymerization and effectively controls the chain transfer reaction during chain growth; it thus achieves fine control over the molecular weight distribution of polymers. This step is crucial for preventing excessive cross-linking or branching, ensuring the uniformity and stability of the polymer structure.

**Figure 3:**
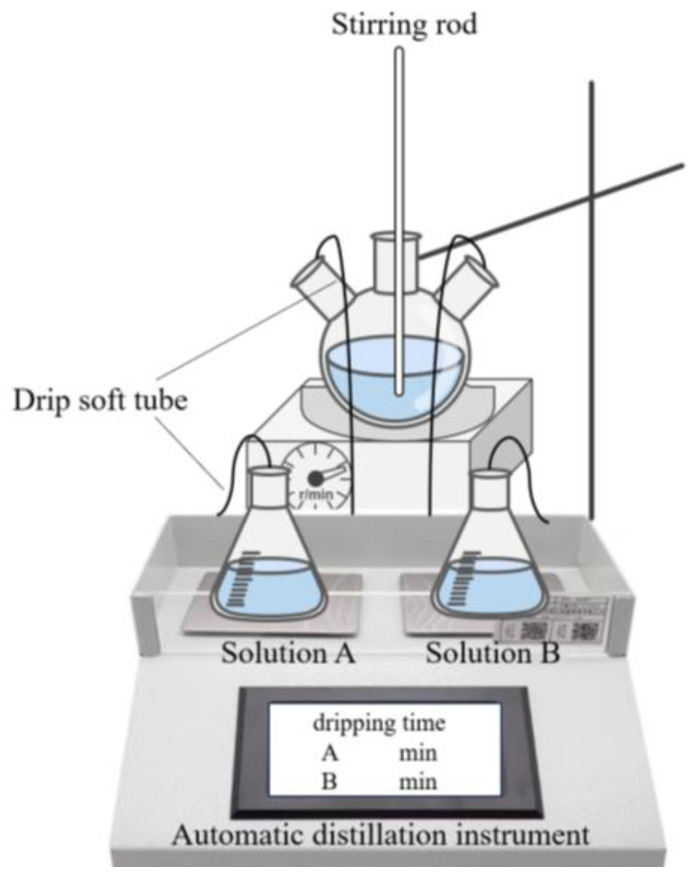
Experimental apparatus for PCE-st synthesis.

Solutions A and B were gradually added at a preset rate into a three-port flask containing a mixture of EPEG and H_2_O_2_ by strictly controlling the key parameters, such as the stirring speed and drop acceleration, for ensuring a smooth and efficient reaction (Figure 3). Upon adding these solutions, the mixture underwent complex radical polymerization in several stages, such as chain initiation, growth, termination, and transfer. Driven by free radicals, PCE-st with AA as the main chain and EPEG and MPEG1-MAA, EPEG2-MAA, and EPEG3-MAA as the branch structure was gradually formed.

Maintaining the ratio of raw materials is crucial during PCE-st synthesis based on the experimental design. The molar ratio of the large monomer (EPEG and MPEG1-MAA, EPEG and MPEG2-MAA, or EPEG and MEG3-MAA) to AA was maintained at 3.5:1 based on an in-depth study and the prediction of the product structure, molecular weight distribution, and properties for optimizing the physical and chemical properties of the polymer. H_2_O_2_ and VC accounted for 1.0% and 0.4% of the mass of all monomers, respectively, which effectively initiated polymerization and protected the reaction system from oxidative degradation, while ensuring polymer stability. MPA accounted for 0.4% of the mass of all monomers, using which PCE-st1, PCE-st2, and PCE-st3 were synthesized with an excellent polymerization degree. (The specific ratio is shown in Table 6).

To ensure stable performances of PCE-st1, PCE-st2, and PCE-st3 and meet usage requirements, 30% sodium hydroxide solution was added to neutralize the pH to 7 ± 1. The precise ratio of these raw materials and additives was adjusted by adding deionized water until the final solid content of the polymer solution reached 40%.

### 3.3. Characterization of Polycarboxylate Superplasticizers

FTIR spectroscopy analysis: The chemical structure of the polycarboxylate superplasticizer was determined via FTIR spectroscopy (Nicol IS50, Thermo Fisher Scientific, Waltham, MA, USA). The polycarboxylate superplasticizer was first dried in an oven and ground into a powder. The background KBr signal was collected before measuring the polycarboxylate superplasticizer content. Finally, 2–3 mg of polycarboxylate superplasticizer samples were mixed with 200 mg of KBr, and FTIR spectra were scanned at 400–4000 cm^−1^.

Zeta potential: The effect of the polycarboxylate superplasticizer on electrostatic repulsion and adsorption properties was determined using the Zeta potential analyzer (Nano ZS90, Malvern Panalytic UK Ltd., London, UK). First, 250 mg of C1 cement was mixed with 100 g of polycarboxylate superplasticizer solution at different dosages (0–1.0%), and the water–cement ratio was 400. After stirring for about 3–5 min, the test was repeated five times, and the average value was taken.

**Figure 4:**
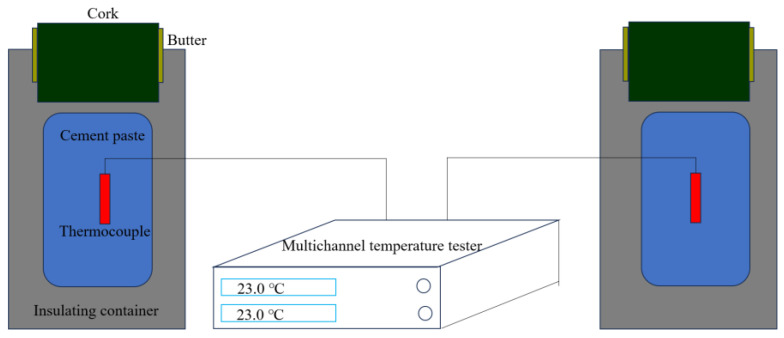
Temperature measurement system of cement paste.

Hydration temperature: A thermocouple was inserted into the cement paste, and the temperature was measured based on the induction principle of thermocouples. The experiment lasted for 48 h (Figure 4). The mass of the C1 cement sample was 300 g, the water–cement ratio was 0.29, and the polycarboxylate superplasticizer content was 0.14%. The ambient temperature was controlled at 23 ± 2 °C.

Fluidity test of cement paste: The fluidity of cement paste was determined according to the GB/T 8077-2023 [19] standard test method for homogeneity of concrete admixtures (C1 cement). The water–cement ratio was 0.29, and the dosage of the water-reducing agent was determined based on the solid value.

Setting time test: According to the GB/T1346-2011 [20] method for testing water consumption, setting time. and stability of cement standard consistency, the setting time of C1 cement was measured at standard-consistency water usage.

Concrete test: The slump test method for concrete mixtures was based on the GB/T 50080-2016 [21] standard test method for the performance of ordinary concrete mixtures. Based on this, the amount of water reducer was determined. Next, standard cube concrete specimens with dimensions of 100 mm × 100 mm × 100 mm were formed using the standard curing method. Each group was composed of three specimens in a standard curing room with relative humidity greater than 95% at 20 ± 2 °C. After curing for 3 d, 7 d, and 28 d, the compressive strength was measured, and the average value was taken.

## 4. Results and Discussion

### 4.1. FTIR Spectroscopy

**Figure 5:**
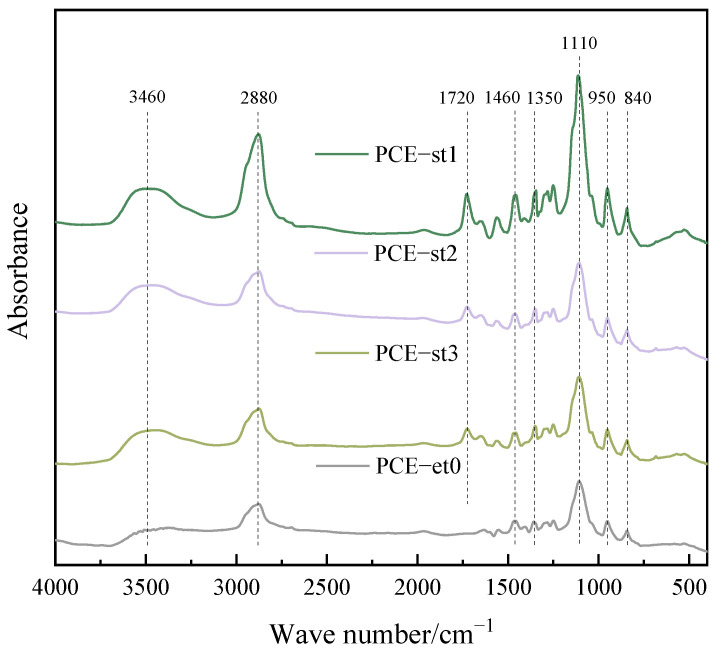
FTIR spectra of polycarboxylate superplasticizers.

Figure 5 shows the FTIR spectrum of a polycarboxylate superplasticizer molecule, which shows a significant and wide wave crest at a wave number of 3460 cm^−1^ [22,23], attributed to –OH vibration in alcohol molecules. Hydrogen atoms in hydroxyl groups are highly reactive and form stable intermolecular hydrogen bond networks with oxygen atoms in neighboring molecules. This strong intermolecular interaction considerably enhances the intensity of –OH stretching vibration and results in a wide distribution of vibration frequencies due to its non-uniformity, forming a wide and strong absorption peak in the spectrum. The adsorption peaks at 2880 cm^−1^ corresponded to the C–H stretching vibration, and the weak absorption peak near 1350 cm^−1^ corresponded to C–C stretching vibration in the main chain [24]. The peaks at 950 and 840 cm^−1^ corresponded to C–OH and C–H tensile vibrations [25], respectively. Moreover, the strong absorption band near 1110 cm^−1^ corresponded to the stretching vibration of the carbon–oxygen bond in C–O–C [26,27]. The ether bond, as an oxygen bridge that bonds two hydrocarbons, connects different functional groups and regulates the molecular conformation of polycarboxylate superplasticizers. Polycarboxylate superplasticizers have a dispersing effect due to the presence of a COOH functional group [28], which is evident from the absorption band at 1460 cm^−1^. These findings revealed the molecular structure and complex chemical bonding states of polycarboxylate superplasticizers.

Characteristic peaks of PCE-st1, PCE-st2, and PCE-st3 were observed near 1720 cm^−1^ that corresponded to C=O stretching vibration [29,30], which is a common feature of functional groups in esters. This indicates that the as-synthesized PCE-st has the structure of an ester. As a bridge connecting organic acids and alcohols, esters enhance molecular stability and positively impact concrete properties, such as reducing concrete sensitivity and improving concrete workability.

**Figure 6:**
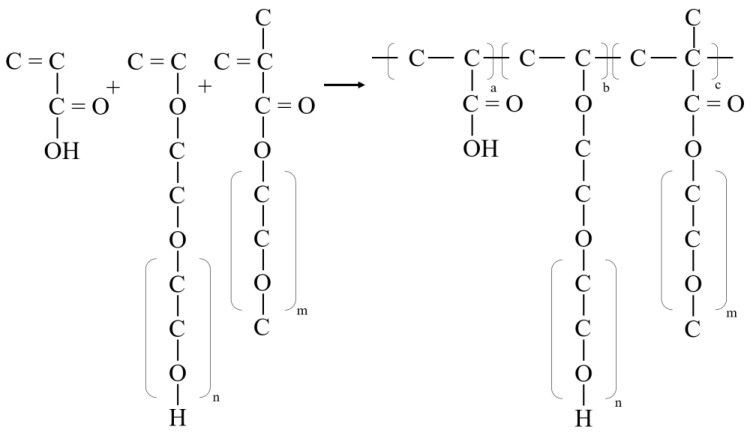
Polymerization reaction formula of PCE-st.

In-depth FTIR analysis revealed the existence and characteristics of key functional groups, such as hydroxyl groups, esters, and ether bonds. These characteristics are fully in line with the original intention and expected effect of the PCE-st design. Figure 6 shows the synthesis and molecular structure of PCE-st.

### 4.2. Zeta Potential Analysis

**Figure 7:**
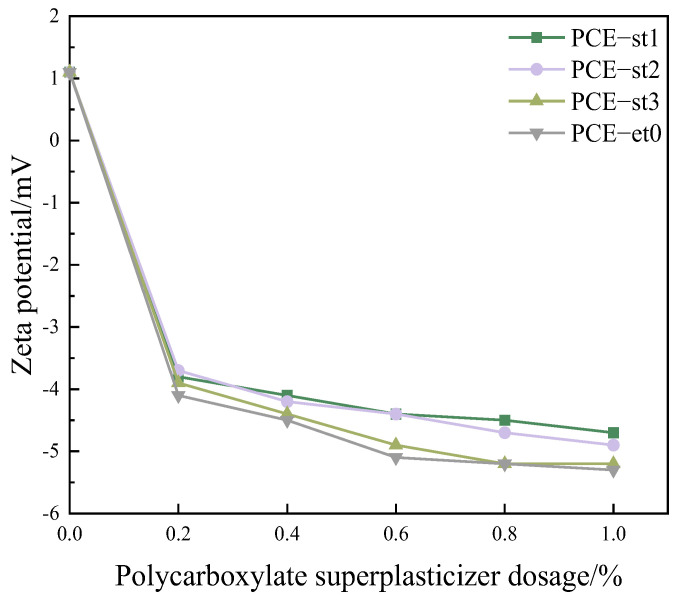
Zeta potentials of cement pastes containing different polycarboxylate superplasticizers.

The zeta potential is a crucial parameter for understanding the adsorption behavior and mechanism of the cement surface. It regulates the cement paste’s performance and optimizes the concrete mix ratio. Therefore, the zeta potential of cement pastes containing various amounts of polycarboxylate superplasticizers (0–1.0%) were systematically and thoroughly studied based on the cement quality (Figure 7).

The zeta potential of the cement pastes rapidly changed from positive (+1.1 mV) to negative upon adding a polycarboxylate superplasticizer. When the polycarboxylate superplasticizer dosage was 0–0.2%, the zeta potential increased by more than 4.8 mV. When the dosage was increased from 0.2% to 1.0%, the absolute zeta potential increased only slightly by 1 mV.

The absolute zeta potential of cement pastes containing the same dosages of PCE-st3 and PCE-et0 was almost similar. Upon adding 0.8% of PCE-st3 and PCE-et0, the zeta potential was −5.2 mV, indicating a consistent dispersion effect of PCE-st1 and PCE-et0. The absolute zeta potential of cement pastes containing PCE-st1 was slightly lower than that of cement pastes containing PCE-st2 and PCE-st3. Carboxyl functional groups and esters can selectively adsorb on the surface of positively charged hydration products (such as calcium hydroxide) formed during cement hydration [31,32,33,34]. As a result, the surface charge properties of the hydrated cement [32,35] changes, and a complex electric double layer is formed around the cement particles. When the molecular weight of the ether remains unchanged, the increase in the weight of MPEG from 400 to 1200 increases the electrostatic force, resulting in an increase in the thickness of the electric double layer. The formation of this layer further aggravates the change in surface charge density and significantly increases the absolute value of the zeta potential.

Upon adding 0.2% of PCE-st1, PCE-st2, and PCE-st3, the difference in the zeta potential of corresponding cement pastes was less than 0.2 mV. Upon adding 0.8% of PCE-st1 and PCE-st3, the difference in the zeta potential of cement pastes was the largest and reached 0.7 mV. As higher amounts of a polycarboxylate superplasticizer are added, the variation in charge density reaches saturation, and the absolute zeta potential reaches a relatively stable value. This process reveals the adsorption dynamic equilibrium of polycarboxylate superplasticizers on the cement particle surface and provides an important theoretical basis for understanding and regulating the dispersion and water-reducing effect of polycarboxylate superplasticizers in cement-based materials [24,36].

### 4.3. Difference in Fluidity Sensitivity of Polycarboxylate Superplasticizers with Varying Water Content

**Figure 8:**
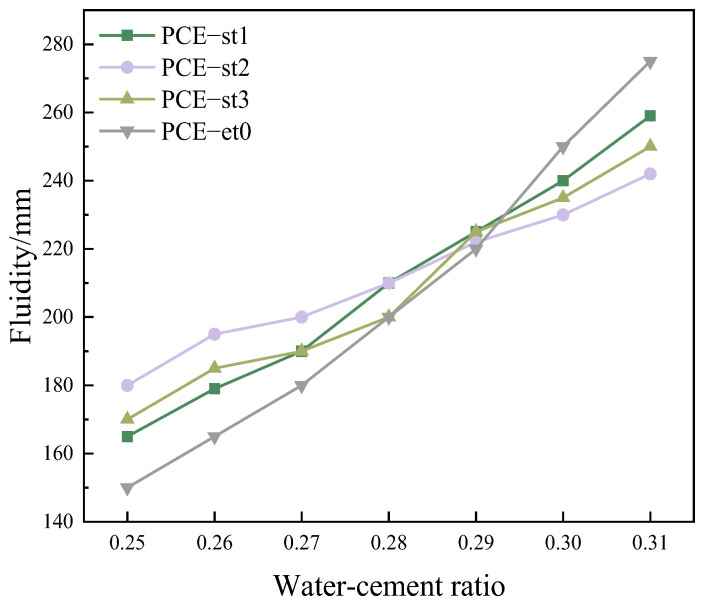
Fluidity sensitivity of different polycarboxylate superplasticizers to varying water–cement ratios (cement pastes).

Figure 8 shows the varying fluidity characteristics of polycarboxylate superplasticizers under different water content variation conditions. The amount of polycarboxylate superplasticizer was adjusted at the beginning of the experiment to stabilize the fluidity of cement pastes within 200 ± 10 mm at a water–cement ratio of 0.28. The influence of variations in water–cement ratios from 0.25 to 0.31 on fluidity was comprehensively studied by determining the corresponding cement pastes’ flow. Results revealed that the fluidity of cement pastes increases with an increasing water–cement ratio.

PCE-et0 was most affected by the water–cement ratio, and the fluidity considerably increased from 150 mm to 275 mm. PCE-st1 and PCE-st3 were moderately affected, with corresponding fluidity increases of 94 mm and 80 mm, respectively. PCE-st2 was least affected by the water–cement ratio, and the fluidity increased slightly from 180 mm to 242 mm. This significant change shows that the fluidity of cement paste is sensitive to the change in the water-cement ratio compared to PCE-st.

The adsorption capacities of PCE-st1, PCE-st2, and PCE-st3 with different molecular weights on the cement particle surface were compared. Owing to its small molecular weight, the adsorption capacity of PCE-st1 on the cement particle surface was relatively weak. Thus, its performance under different water content variation conditions was not sufficiently stable, with high sensitivity to water content variations. In contrast, PCE-st2, with a medium molecular weight and a unique ester-based structure in alkaline hydrolysis, could effectively regulate the flow of cement pastes and exhibited lower sensitivity to water content variations. However, PCE-st3, with a large molecular weight, was less soluble and unevenly dispersed in the cement pastes, exhibiting enhanced sensitivity to water content variations.

### 4.4. Fluidity Sensitivity of Different Polycarboxylate Superplasticizers with Different Cements

**Figure 9:**
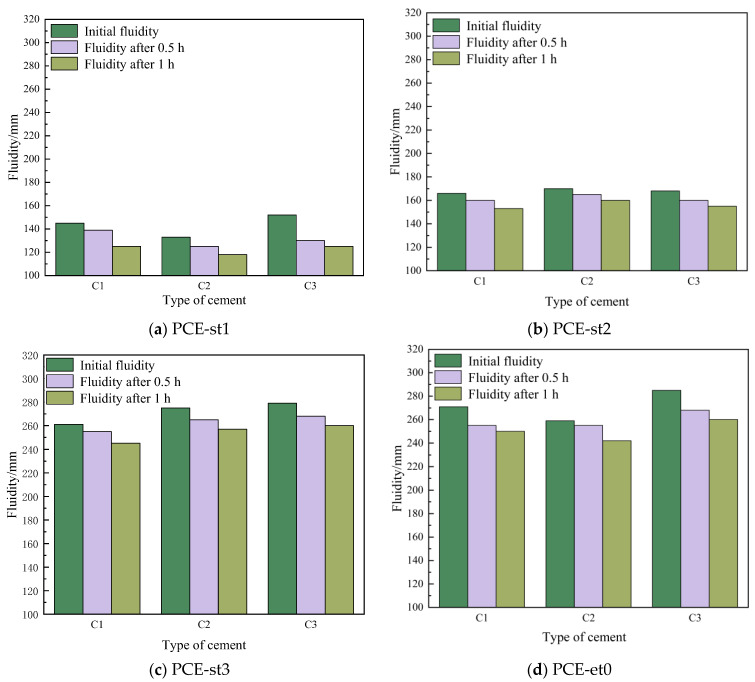
Influence of different polycarboxylate superplasticizers on the fluidity of different cement pastes.

As shown in Figure 9, the fluidity difference of PCE-et0 in different cements was the largest, indicating that PCE-et0 is the most sensitive to cement differences. In PCE-st, the fluidity difference of PCE-st2 in different cements was the smallest, indicating that PCE-st2 is the least sensitive to cement differences. These experimental results were also consistent with the conclusion of water content variation sensitivity.

**Figure 10:**
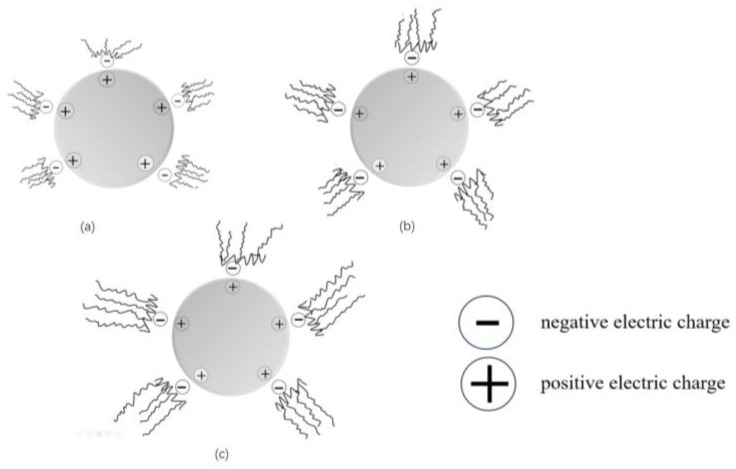
Schematic of the working mechanism of PCE-st: (**a**) PCE-st1, (**b**) PCE-st2, and (**c**) PCE-st3.

PCE-st exhibited good dispersibility in all cement pastes. When the PCE-st content was 0.14%, the initial fluidity reached 133~279 mm. This was primarily because the specific adsorption groups in PCE-st adhered closely to the cement particle surface, whereas its side chain was fully extended in the liquid phase of the cement pastes to form a polymer film with a certain thickness. This film effectively prevented the mutual aggregation of cement particles, promoted their uniform dispersion, and considerably improved the initial fluidity of cement pastes. Among them, C1, C2, and C3 containing PCE-st3 showed the best initial fluidity of 261–275 mm. This was mainly because PCE-st3 had the largest molecular weight and the longest side chain, which enabled its strongest adsorption capacity when in contact with cement particles [37,38]. This strong adsorption and steric hindrance allowed the cement particles to disperse more easily, considerably improving the fluidity of cement pastes [39] (Figure 10).

PCE-st1 and PCE-st3 compared to PCE-et0. PCE-st3 exhibited good plasticity retention, and its corresponding cement pastes exhibited a fluidity loss of 14.3% and 6.0% after 1.0 h of hydration. This was mainly because more ester groups in PCE-st3 underwent hydrolysis in the alkaline cement paste environment [40] and formed dispersed carboxylate particles. This chemical change further enhanced the dispersion of PCE-st and considerably extended the fluidity retention time. The cement pastes maintained high fluidity even after mixing for 0.5 h or 1.0 h, which is crucial for improving the workability of concrete.

A comparison of cement pastes flows before and after the addition of PCE-st revealed the general applicability of PCE-st in various cement types. Irrespective of the cement type, PCE-st can stably and reliably improve the dispersibility and plasticity retention of cement.

### 4.5. Influence of Hydration Temperature on Cement Pastes Containing Different Polycarboxylate Superplasticizers

**Figure 11:**
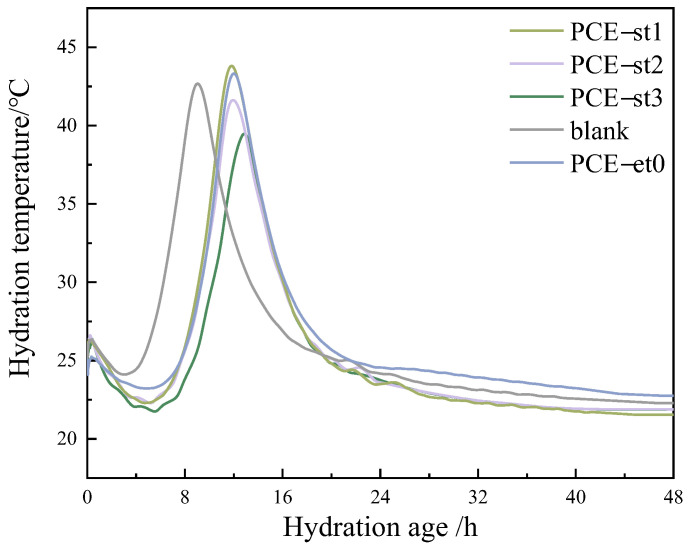
Hydration temperatures of different polycarboxylate superplasticizers.

The cement hydration temperature must be closely monitored during the construction of high-strength concrete. A temperature stress exceeding the tensile strength of concrete will cause concrete cracking and affect the integrity and durability of the structure [4,41]. Figure 11 shows the changes in hydration temperature with time for cement pastes without polycarboxylate superplasticizers and those containing 0.14% of PCE-st and PCE-et0.

Figure 11 shows that the temperature of all samples increased by about 1.0 °C after 0.3 h of hydration. The temperaturereached its lowest value after 5 h of hydration (2.8 h for the blank group without the polycarboxylate superplasticizer). This indicated that polycarboxylate superplasticizers delay cement hydration. At this point, cement hydration was in the induction stage and almost stopped. The hydration of cement containing polycarboxylate superplasticizers was delayed mainly due to the adsorption and coating effect of the polycarboxylate superplasticizers and the retarding properties of carboxylic and hydroxyl groups in their main chain structures. A comparison of PCE-st1, PCE-st2, and PCE-st3 revealed that higher molecular weights profoundly delay and inhibit cement hydration because of the high hydrolysis degree of ester groups.

As cement hydration continued, the hydration temperature of the blank group without polycarboxylate superplasticizers first reached a peak of 42.7 °C after 9.0 h. Compared to the blank group, although PCE-et0 can delay the hydration of cement, the peak value of hydration temperature slightly increases, which indicates that although the carboxyl functional groups in PCE-et0 can coordinate with calcium ions in cement to form complexes, these complexes may reduce the concentration of ions in the solution, thus slowing down the nucleation and growth rate of hydration products. However, once the hydration reaction begins, the presence of a water reducer may change the surface properties and reaction path of cement particles, resulting in more concentrated and intense reaction heat release. Therefore, the heat released during the same time may be more, so the hydration temperature increases.

A comparison of the hydration temperatures of cement pastes containing PCE-st1, PCE-st2, and PCE-st3 revealed that PCE-st3 was the last to reach its peak hydration temperature. Moreover, it delayed the hydration time from 11.9 h to 12.9 h compared to PCE-st1; however, the delay time of PCE-st3 reached 4.0 h compared to the blank group. The peak hydration temperature of PCE-st3 decreased considerably and reached its peak of only 39.5 °C, considerably lower than that of PCE-st1 (43.8 °C). After about 40 h, the hydration temperature stabilized, and cement hydration ceased. This could be due to the different reaction modes of ester, ether, and hydroxide groups in cement. Esters easily react with hydroxides to form colloidal substances that tightly bind to the cement particle surface and form a film. This phenomenon reduces the direct contact and reaction rate between cement particles and water, thereby extending the hydration time and enabling uniform heat release due to cement hydration. As a result, substantial heat accumulation in a short time is prevented.

### 4.6. Influence of Different Polycarboxylate Superplasticizers on the Setting Time of Cement

**Figure 12:**
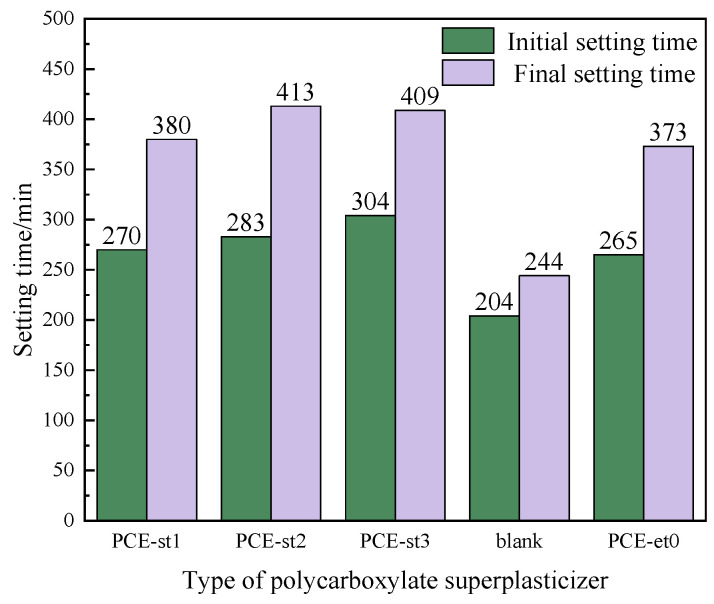
Influence of different polycarboxylate superplasticizers on the cement-setting time.

The effect of polycarboxylate superplasticizers on the setting time of cement pastes is a complex and fine process, involving several aspects, such as molecular interaction, cement hydration dynamics, and microstructural evolution. Figure 12 shows the difference in the initial and final setting times of cement pastes by precisely controlling the amounts of PCE-st1, PCE-st2, and PCE-st3 at a constant water–cement ratio of 0.29.

After adding PCE-et0 or PCE-st, the initial setting time could be extended from 204 min to 265 min~304 min. This phenomenon can be attributed to the molecular structure of PCE-et0 or PCE-st and its interaction with cement hydration products. In particular, the carboxyl functional group in PCE-et0 or PCE-st acts as an active site for efficient complexation with Ca^2+^ in the cement pastes and effectively reduces the concentration of free Ca^2+^, and the formation rate of C–S–H gel decreases. Therefore, the setting time of cement is prolonged [42,43].

An analysis of the difference in the initial setting times of PCE-st1, PCE-st2, and PCE-st3 revealed the important influence of their molecular structure on performance. Due to the large molecular size of PCE-st3, it did not penetrate the tiny pores of cement particles. However, it strongly adsorbed on the particle surface and formed a lubricating film, which prevented direct contact with cement particles and prolonged the initial setting time. In contrast, PCE-st1, with its smaller molecular weight and excellent permeability, penetrated deeper into the cement particles and effectively dispersed, while reducing agglomeration, because in this form, this formulation creates the impression of an accelerating admixture.

The final setting time of PCE-et0 (373 min) was shorter than that of the cement containing PCE-st, primarily due to the retarding effect of C=O in PCE-st [44]. The final setting time of PCE-st1 was 380 min, considerably lower than that of PCE-st2 and PCE-st3 (413 min and 409 min, respectively). These observations further confirmed that the strong permeability of PCE-st1 promotes the uniform dispersion of cement particles and accelerates the hydration reaction by optimizing the cement microstructure. Due to the difference in the molecular characteristics of PCE-st, the penetration and dispersion effects of PCE-st2 and PCE-st3 were slightly worse, and the final solidification time of their corresponding cement pastes was relatively long.

### 4.7. Influence of Polycarboxylate Superplasticizers on the Workability and Mechanical Properties of Concrete

**Figure 13:**
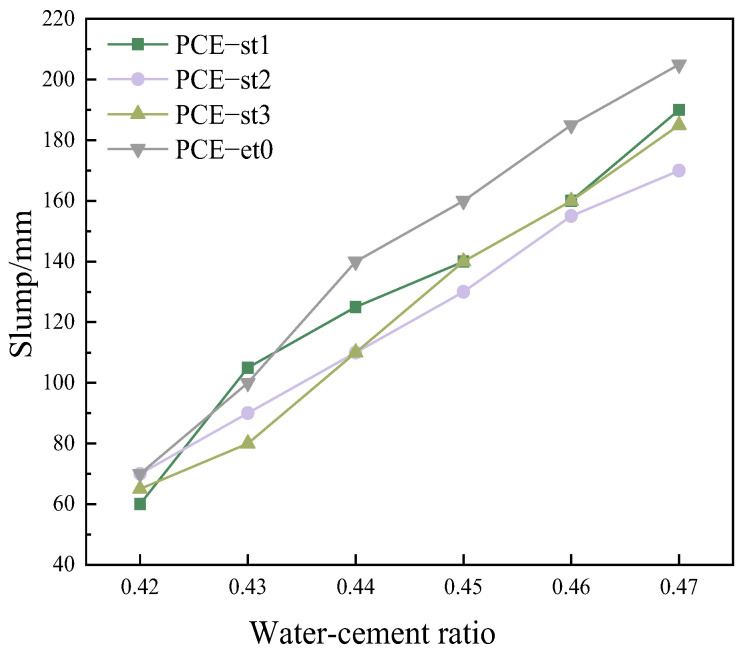
Slumpsensitivity of different polycarboxylate superplasticizers to varying water–cement ratios (concrete).

During high-strength concrete production, its sensitivity to fluctuations in concrete mixing water is particularly prominent. This property directly and profoundly affects the workability of concrete, as well as other parameters. The slump value of high-strength concrete at a water–cement ratio of 0.42–0.47 was determined. Figure 13 shows the positive effect of PCE-st on reducing the sensitivity of cement to fluctuations in water–cement ratios. The slump value increased from 70 mm to 205 mm for cement containing PCE-et0, whereas that of cement containing PCE-st1 and PCE-st3 increased by 130 mm and 120 mm, respectively. However, the slump for PCE-st2 increased by only 100 mm, from 70 mm to 170 mm. Moreover, PCE-st2 had the most significant impact on reducing the sensitivity of cement to variations in the water–cement ratio, consistent with the cement paste experiment.

Mechanical properties play a crucial role in the in-depth and systematic study of concrete properties [45,46]. Herein, the slump range was set to 70 ± 5 mm for simulating the concrete-pouring conditions on-site and ensuring that the experimental data were almost similar to the actual application scenario.

**Figure 14:**
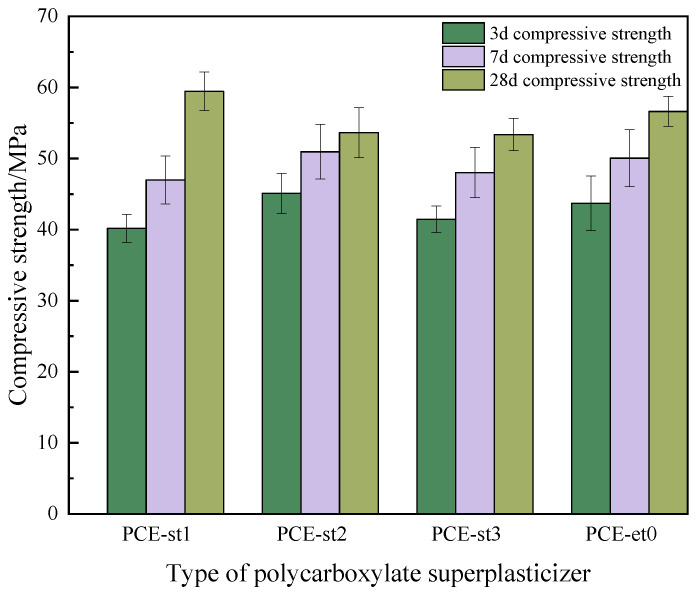
Compressive strength of concrete with different polycarboxylate superplasticizers.

The effects of PCE-st on the compressive strength of concrete were experimentally investigated (Figure 14). Concrete mixed with PCE-st2 and PCE-st3 exhibited the best workability (Table 7). Moreover, PCE-st3 exhibited the best slump retention performance because of the presence of esters, a larger molecular weight, and a higher hydrolysis degree. The lowest and highest compressive strengths of concrete containing PCE-st after curing for 28 d could reach at least 53.7 MPa and 59.4 MPa, respectively. These were comparable to the 28 d compressive strength (56.6 MPa) of commercial PCE-et0-mixed concrete.

## 5. Conclusions

In this study, MPEG1-MAA, MPEG2-MAA, and MPEG3-MAA were synthesized by esterifying MAA with MPEG1 (400), MPEG2 (600), and MPEG3 (1200). These were polymerized with EPEG and AA to yield PCE-st1, PCE-st2, and PCE-st3. These were analyzed via infrared spectroscopy and zeta potential measurements. Results revealed that these PCE-sts contain ester and ether bonds in their molecular configurations; moreover, their zeta potentials are lower, and they exhibit good adsorption performance. The effects of MPEG with different molecular weights on cement pastes and concrete were compared. The main conclusions are as follows:

(1) As the MPEG weight of the ester monomer increases, the sensitivity of cement to water content variations first decreases and then increases. As the water–cement ratio increases from 0.25 to 0.31, the fluidity of cement pastes mixed with PCE-st2 only increases by 62 mm, and that of cement pastes mixed with PCE-st1 and PCE-st3 increases by 94 and 80 mm, respectively.
(2) The fluidity retention of cement pastes increases with increasing weight of MPEG. The flow loss of cement pastes mixed with PCE-st3 at 1 h is 6.0%, which is less than that of cement pastes mixed with PCE-st1 (14.3%).
(3) Compared to the commercially available PCE-0, the synthesized PCE-st considerably delays cement hydration and reduces the peak hydration temperature. When the molecular weight of MPEG increases from 400 to 1200, the hydration temperature decreases by 10%, and the final setting time prolonged by 7.6%.
(4) The synthesized PCE-st has excellent construction workability, and the compressive strength of concrete can be as high as 59.4 MPa after 28 d of standard curing, which is comparable to the compressive strength (56.6 MPa) of concrete prepared by commercially available PCE-et0 under the same curing conditions.

## Figures and Tables

**Table 1:** Main chemicals used in the experiments.

Chemical Raw Materials and Reagents	Specification	Manufacturer
Ethylene glycol polyethylene glycol ether (EPEG = 3000)	Industrial grade	Liaoning Xindun Pharmaceutical Chemical Co., Ltd. (Liaoning, China)
Methoxy polyethylene glycol ether (MPEG1 = 400, MPEG2 = 600, and MPEG3 = 1200)	Industrial grade	Shandong Yousuo Chemical Technology Co., Ltd. (Shandong, China)
Methacrylic acid (MAA)	Analytical reagent	Jinan Haokun Chemical Co., Ltd. (Shandong, China)
P-toluene sulfonic acid (PTSA)	Analytical reagent	Guangzhou Shanghe Chemical Technology Co., Ltd. (Guangdong, China)
Hydroquinone (HQ)	Analytical reagent	Guangzhou Shanghe Chemical Technology Co., Ltd. (Guangdong, China)
Cyclohexane (CH)	Analytical reagent	Guangzhou Shanghe Chemical Technology Co., Ltd. (Guangdong, China)
Acrylic acid (AA)	Analytical reagent	Jinan Haokun Chemical Co., Ltd. (Shandong, China)
Ascorbic acid (VC)	Analytical reagent	Jiangsu Aofu Biotechnology Co., Ltd. (Jiangsu, China)
Hydrogen peroxide solution (H_2_O_2_)	28% concentration	Guangdong Shangshan Chemical Co., Ltd. (Guangdong, China)
Mercaptopropionic acid (MPA)	Analytical reagent	Guangzhou Shanghe Chemical Technology Co., Ltd. (Guangdong, China)
Sodium hydroxide (NaOH)	Analytical reagent	Guangzhou Shanghe Chemical Technology Co., Ltd. (Guangdong, China)
Polycarboxylate superplasticizer (PCE-et0)	Industrial grade	Shaanxi Kezhijie New Material Co., Ltd. (Shaanxi, China)

**Table 2:** Chemical compositions of cement (%).

Cement	CaO	Al_2_O_3_	SiO_2_	Fe_2_O_3_	SO_3_	Na_2_O	K_2_O	Loss	Others
C1	60.20	4.50	23.81	3.33	2.33	0.56	0.20	1.58	3.49
C2	59.70	4.40	22.40	3.27	2.28	0.39	0.31	1.48	5.77
C3	59.71	4.59	22.56	3.11	2.01	0.23	0.51	1.60	5.68

**Table 3:** Mechanical properties of mortar.

	Strength	Flexural Strength (MPa)			Compressive Strength (MPa)		
Cement		3 d	7 d	28 d	3 d	7 d	28 d
C1		5.8	6.5	7.3	28.2	36.5	45.0
C2		5.9	6.1	7.4	27.3	35.8	45.2
C3		6.2	6.1	7.5	30.3	37.4	48.3

**Table 4:** Particle sizes of aggregates.

Aggregate	Particle Size (mm)
River sand	0.25–0.35
Machine-made sand 1	0.35–0.65
Machine-made sand 2	1.20–2.20
Small stone	4.80–9.50
Large stone	9.50–19.0

**Table 5:** Proportions of concrete.

Cement (kg/m^3^)	Water–Cement Ratio	River Sand (kg/m^3^)	Machine-Made Sand 1 (kg/m^3^)	Machine-Made Sand 2 (kg/m^3^)	Small Stone (kg/m^3^)	Large Stone (kg/m^3^)
360	0.42	400	66	200	800	430

**Table 6:** Different formulations of PCE-st.

	n (EPEG and MPEG1-MAA):nAA	(EPEG and MPEG2-MAA):nAA	n(EPEG and MPEG3-MAA):nAA	H_2_O_2_	VC	MPA
PCE-st1	3.5:1	0	0	1.0%	0.4%	0.4%
PCE-st1	0	3.5:1	0	1.0%	0.4%	0.4%
PCE-st1	0	0	3.5:1	1.0%	0.4%	0.4%

**Table 7:** Influence of different polycarboxylate superplasticizers on the compressive strength of concrete.

	Cohesiveness	Slump (mm)	
		0 h	1 h
PCE-st1	Normal	75	55
PCE-st2	Excellent	70	55
PCE-st3	Excellent	65	55
PCE-et0	Normal	70	50

## Data Availability

The original contributions presented in this study are included in the article. Further inquiries can be directed to the corresponding author.
